# The Last Month of the Year

**Published:** 1898-12

**Authors:** 


### Editorial.

### THE LAST MONTH OF THE YEAR.

THE last month in the year naturally marshals the thoughts of men and women into order and reflection. Exactly why this should be, one day, one month, or one year, more than another, is a problem of the human mind not easily explained. The sentimental side of human nature is always paramount, and probably is the outweighing force that leads the thoughts of most people into the reflective vein on such occasions. Yet there is a side to this retrospection beyond sentimentalism, and one that belongs more nearly to the outgrowth of humanity towards higher planes of active thought and practice.

It broadens the mind to drop for a time local interests and local contentions and expand the view over the world's activities. The study of history enlarges the horizon of every person, and is, in a minor degree, the equivalent to extended travel, than which there is nothing better to enlarge the sphere of thought, and to bring the self-opinionated and egoistic man to an appreciation of his limitations and narrowness.

Thus each year brings with it the reflective period, and as we come to the last month of the year 1898, the feeling is impressively present that the year 1900 will soon be with us, closing the century, and will terminate one hundred years of great progress in every direction.

Dentistry has much to be thankful for in a retrospect of these years, for this century has witnessed its development from the crudest beginnings to a position worthy the respect of all men.

While we may with gratification look back over the past, the wise thinker will not dwell thereon. The past is always safe. Like our dead loves in the neighboring cemetery, we need feel no concern for their cast-off organisms, but the living spirit that animated these is of ever-increasing interest. So the dead past is an educational memory, but the vital interest lies not there, but in the activities of the present and in the hopes of the future.

The year has been one of deep anxiety and sorrow to many households. Youth has with generous enthusiasm gone out to battle, and the graves of the martyrs have been more than filled. Time can alone determine whether the sacrifice of blood and treasure will be worthy the close of the century. War is a relic of our barbaric instincts. It is the crude method of the savage to establish his authority. To the thinking man or woman this killing of men to advance the civilization of the world seems a moral paradox, yet, as far as our limited vision will enable us to peer into the unknown, it will remain the most potent method in establishing "Peace on earth and good-will to men." It means eventually the "survival of the fittest," and then will come upon the ruins of empires the period when "swords will be beaten into ploughshares."

The progress of dentistry the past year has not been remarkable. No startling innovations in theory or practice have been made. There has been activity, but it has not resulted in any modification of practice. Much was hoped from the college association known as The National Association of Dental Faculties. The work performed by this body has, it is surmised, come up to expectations. It is leading the educational thought into correct channels, and can be depended upon to do this in the future, providing it crushes professional politics in its incipiency. The caucus has no place in this body, and, it may be hoped, will never get within the bounds of the organization.

The professional portion of the world in this country, knows that the National Dental Association was not satisfactory. It is not difficult to find fault, but an explanation for the failure is not so readily found. The war, heat, and a score of other reasons have been given, but they do not meet the real explanation in the estimation of the writer, and this lies in the fact that localities have so changed the method of former years that State, interstate, and local society combinations have centralized the interest to such a degree that the same men who will work enthusiastically for these subordinate bodies will not care to repeat the effort for an association meeting but once a year, and it may be a thousand miles from home. Dentistry needs an association of a different character. The writer outlined his views in the address before the last meeting of the American Dental Association, and he has nothing to add thereto. The central body should be the distinguishing body, composed of the best elements of all below it. When this is accomplished, men will travel across the continent to have the honor of uniting in its deliberations.

While there has been much to discourage in the past twelve months, the failures give the promise of a better outlook for the future. Individuals, societies, professions, all grow by their failures, providing the sufferers are made of that stern material that is ever ready to build the new house on the ruins of the old, and make it more worthy the period. Thus the past year, with its glories and mistakes, may become the indicator of progress to be held in worthy remembrance by those who succeed us. The year 1898, therefore, while not prolific in much for dentistry, has a value for those who are able to read the lessons of the years, as they come and go, and profit thereby.

